# Design Optimization Method for Large-Size Sidewall-Driven Micromixer to Generate Powerful Swirling Flow

**DOI:** 10.3390/mi14122246

**Published:** 2023-12-16

**Authors:** Daichi Yamamoto, Toshio Takayama

**Affiliations:** Department of Mechanical Engineering, Tokyo Institute of Technology, 2-12-1, Ookayama, Tokyo 152-8552, Japan

**Keywords:** micromixer, pressure vibration, spheroid, drug discovery

## Abstract

Microfluidic devices, which miniaturize cell culture and chemical experiments from lab-scale to microchip dimensions, have gained significant attention in recent years. Extensive research has been conducted on microfluidic mixers, which facilitate the mixing and agitation of chemicals. The “Sidewall-Driven Micromixer” that we are currently developing employs a unique mechanism; it induces a swirling flow within the main chamber by vibrating the silicone wall situated between the main and driving chambers using pressure fluctuations. In an earlier study, we found that Sidewall-Driven Micromixers of a size suitable for small cells could indeed produce this swirling flow. Furthermore, we successfully established concentration gradients within each mixer. However, when attempting to upscale the mixer while maintaining conventional proportions to accommodate larger cell aggregates such as spheroids, the desired swirling flow was not achieved. To address this challenge, we made adjustments to the wall dimensions, aiming to amplify wall deformation and thereby enhance the mixer’s driving force. Concurrently, we modified the mixer’s shape to ensure that the increased wall deformation would not hinder the fluid flow. These alterations not only improved the mixer’s performance but also provided valuable insights for positioning the mixer’s neck channel, considering the extent of wall deformation.

## 1. Introduction

**Figure 1:**
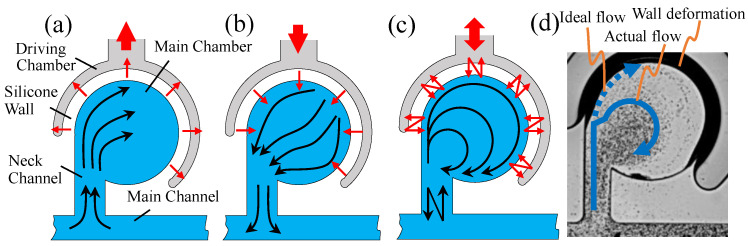
Mixing principle of sidewall-driven micromixer: (**a**) shows the streamline under wall-pulling phase, and (**b**) shows the streamline under wall-pushing phase, respectively. These two phases are repeated to generate the swirling flow in the main chamber as shown in (**c**). (**d**) shows the state when wall deformation obstructs the flow along the wall.

Cell culture and chemical experiments have been traditionally conducted in large vessels such as Petri dishes, thus necessitating significant space even for tiny samples and producing considerable amounts of waste fluid. Conversely, recent advancements have led to the development of microfluidic devices, which allow these experiments to transition from the expansive laboratory setting to compact microchips. There has been a surge in research around microfluidic mixers, devices designed for the mixing and agitation of various substances, including drugs and cells. Passive mixers, for instance, have been explored extensively [1,2,3,4,5,6]. These mixers increase the contact area between two liquids to enhance diffusion by innovatively shaping the flow path. For example, Che-Hsin Lin et al. enabled two-component mixing by generating three-dimensional vortices in a circular chamber with fluid self-rotation [7], and Chien-Chong Hong et al. enabled mixing using a Tesla valve structure [8]. In addition, Jing-Tang Yang et al. developed a passive mixer that enables the mixing of liquids by creating grooves in the flow channel [9]. Vladimir Viktorov et al. developed a 3D splitting and recombination (SAR) passive micromixer with microstructures placed on the top and bottom floors of micro channels called a chain mixer [10]. On the other hand, active mixers have garnered attention for their ability to force-mix two liquids through mechanisms such as electro-osmosis [11] and acoustic vibrations [12], among others [13,14,15,16,17,18]. For instance, Naresh Veldurthi et al. developed a micromixer with a magnetic actuator. They used a magnetic stirrer to rotate a micro-rotor, which generated a vortex in the chamber and enabled the mixing of two liquids [19]. Po-Hsun Huang et al. developed a mixer that uses acoustic vibration to generate vortices at the edges of the flow channel [20]. M. H. Oddy et al. were able to mix two liquids by initiating a flow instability, which they had observed in sinusoidally oscillating, electro-osmotic channel flows [21]. Our contribution to this field is the introduction of the “Sidewall-Driven Micromixer” as a type of active mixer. The driving principle behind this mixer is depicted in Figure 1. Structurally, the mixer consists of a main chamber connected to a main channel via a neck channel, and the main chamber is placed inside an encircling driving chamber. There is no direct flow channel connection between the two chambers. Pressure variations introduced to the driving chamber cause the silicone wall to oscillate and deform, transmitting force to the main chamber in the process. During the wall-pulling phase, as shown in Figure 1a, the main chamber expands, drawing liquid from the main channel, which then follows the main chamber’s sidewalls. Conversely, in the wall-pushing phase, illustrated in Figure 1b, the main chamber contracts, expelling the liquid back into the main channel. As illustrated in Figure 1c, these alternating flow patterns promote an asymmetrical liquid exchange between the main chamber and the main channel, generating a swirling flow within the main chamber. This mechanism enables efficient mixing of the liquid from the main channel with the contents of the main chamber, ensuring thorough agitation within the chamber. In previous studies, the stirring was made possible by a driving method that applied oscillatory pressure to the main channel with a similar geometry, but with that method, when multiple main chambers are connected, mixing occurs simultaneously in all chambers. Therefore, we developed the “side-wall-driven type” in which the main chambers are surrounded by driving chambers, aiming to drive multiple mixers independently. In fact, by driving them with different pressure distributions, they succeeded in creating a different concentration in each main chamber.

The Sidewall-Driven Micromixer not only mixes liquids, but also maintains a constant concentration of liquid in the main chamber by injecting air into the main channel, and agitates only the liquids in the main chamber. Using this mechanism, cells can be trapped in the main chamber. Therefore, this mixer is suitable for use in cellular experiments. In this study, we propose a new micromixer that can mix spheroids and drugs based on the principle of a Sidewall-Driven Micromixer. Spheroids are spheroidal aggregates of cells in three-dimensional culture. Spheroids can reproduce the tissue conditions of a disease. Given this property, spheroids have been proposed for use in screening tests to confirm drug efficacy [22,23]. When spheroids and drugs are mixed in a conventional Sidewall-Driven Micromixer, the size of the mixer poses a challenge. The spheroids that are larger than conventional micromixers, with a neck channel width of 50 µm and channel height of 100 µm, cannot be inserted into the mixer. For example, the spheroids of HeLa cells are in the range of 70–170 µm in size [24]. Hepatocyte spheroids with high cell viability and no oxygen limitation are up to 100 µm in diameter [25]. Therefore, we tried to increase the size of the Sidewall-Driven Micromixer to enable the insertion of spheroids while maintaining the conventional dimensional ratio. However, this resulted in a decline in mixing efficacy. We postulate that this reduction was due to the relatively minimal wall deformation arising from the mixer’s increased size, subsequently decreasing the volume of liquid drawn into the main chamber. To address this, we considered modifying the aspect ratio by increasing the wall height relative to its thickness, aiming to enhance the silicone wall’s deformation. Furthermore, we transitioned from a piezoelectric actuator to air as our pressure vibration source. Although piezoelectric actuators can achieve high-frequency liquid injections and ejections using a syringe pump, their amplitude is limited. When channeled through a constricted channel, the pressure delivered to the driving chamber experiences significant dampening. Hence, the liquid flux in and out of the driving chamber becomes inadequate, especially for a chamber of considerable volume. Conversely, although air pressure operates at a lower frequency, its potential to induce substantial wall deformations is noteworthy. These modifications indeed augmented the mixing performance of larger mixers. Nonetheless, we encountered a new challenge. As depicted in Figure 1d, significant wall deformations obstructed the flow along the main chamber’s wall during driving, leading to suboptimal mixing. This study delves into determining the ideal parameters for a large Sidewall-Driven Micromixer, considering the wall deformations induced by air pressure. Furthermore, we demonstrate the enhanced mixing capabilities of the developed mixer.

## 2. Materials and Methods

### 2.1. Fabrication Method for the Sidewall-Driven Micromixers

The Sidewall-Driven Micromixer is fabricated using photolithography. The photoresist was SU8-3050 (KAYAKU Advanced Materials, West Borough, MA, USA), and the mold was fabricated using a maskless photolithography system (PALET; NEOARK Corporation, Tokyo, Japan). Previously, the molds were fabricated by the photolithography using masks, but this study utilized a maskless system to easily enable high aspect ratios. PDMS (SILPOT184; Dow, Midland, MI, USA; main agent:hardener = 10:1 (mass ratio)) was poured into the mold and cured to transfer the pattern. The PDMS, on which the flow channel pattern was transferred, was bonded to a glass slide by plasma surface treatment. A flow channel was formed during these steps. We considered increasing the driving force by reducing the amount of curing agent and softening the silicone, but we observed that an extremely low amount of curing agent caused poor curing and the silicone became sticky. It is thought that the liquid silicone that did not react with the curing agent remains and leaks into the flow channel. Therefore, to change the driving force, we thought it would be more reproducible to change the aspect ratio of the wall or the driving pressure. The method of reducing the curing agent was not used in this experiment. Moreover, if the ratio of the curing agent is changed, both parameters, dimensions and elasticity, will change, making discussion of the results difficult. Therefore, in this study, only dimensions were changed.

### 2.2. Experimental Setup

**Figure 2:**
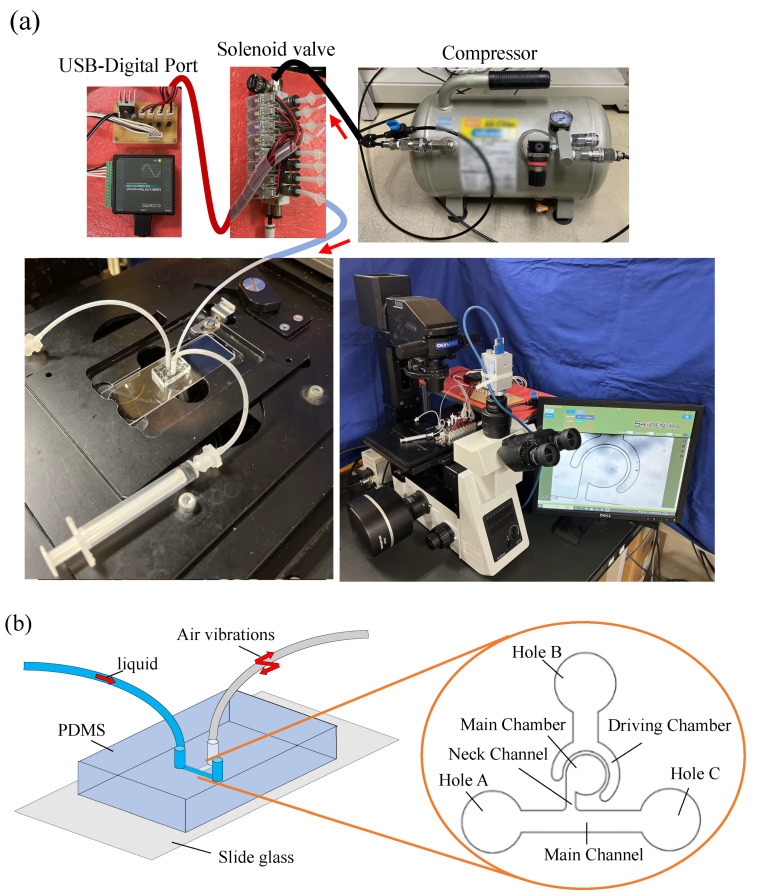
Experimental setup, (**a**) shows actual equipment, including mixer driving units, (**b**) shows the names of the Sidewall-Driven Micromixer’s parts.

**Figure 3:**
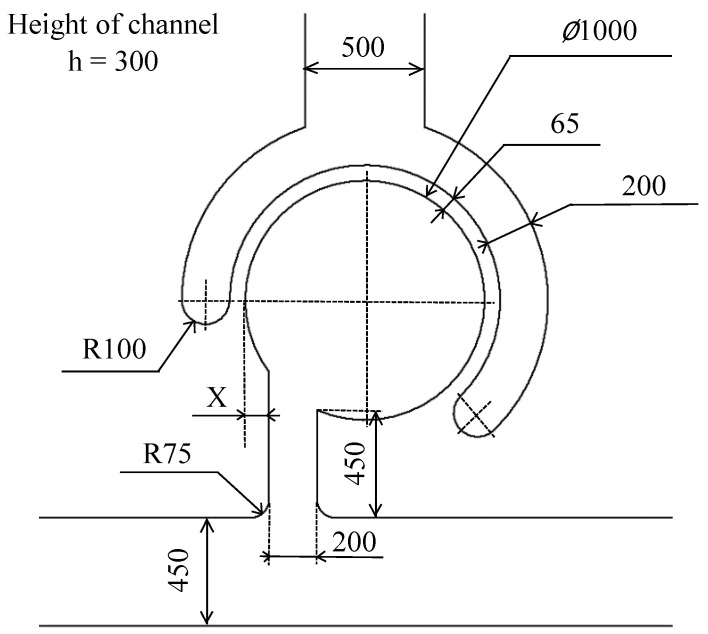
Dimensions of Sidewall-Driven Micromixer in this study.

Figure 2a shows the experimental setup. Air pressure vibrations are supplied by a compressor (AK-T20R, Max, Inc., Tokyo, Japan) to the driving chamber of the mixer via a solenoid valve. The solenoid valve is controlled using a USB-connected digital input/output device (DO-16TY-USB, Contec Corporation, Osaka, Japan). A syringe is used to inject liquid with 3-µm microbeads (Polybead Polystyrene 3.0 Microspheres; Polysciences, Philadelphia, PA, USA) into the main channel of the mixer, and the movement of the microbeads during mixer driving is observed via a microscope (IX73P1F; OLYMPUS, Tokyo, Japan). The movement of the microbeads is captured using a high-speed camera (CHU130EX; SHODENSHA, Tokyo, Japan). In this study, the frame rate of the camera is 500 fps, and the shutter speed is 1/10,000 s. The PDMS chip has a flow channel in the shape shown in Figure 2b, and each of the three holes is perforated such that a tube can be inserted into it. The liquid with the microbeads is injected into hole A from the syringe, and the air pressure vibration is sent to hole B from the solenoid valve. Air pressure vibration drives the mixer. Hole C is used to discharge the waste liquid. Figure 3 shows the dimensions of the mixer used in this study. In this design, the width of the main channel is 450 µm, the width of the neck channel is 200 µm, the diameter of the main chamber is 1000 µm, and the channel height is 300 µm, considering that spheroids in the range of 100–200 µm in size can be inserted. In previous studies, X was 0 µm (the neck channel was tangential to the main chamber). However, in this study, variable X is used to compare different neck channel positions for reasons described later. The aspect ratio of the silicone wall should be as large as possible to increase wall deformation and driving force. Based on the performance of the maskless photolithography system, an aspect ratio of 4.6 is determined to be the maximum and the one can be made most stably. The wall thickness is designed to match this value. The wall thickness is 65 µm, and the wall height is 300 µm, which is equal to the channel height; thus, the aspect ratio is 4.6.

In a previous study in our own lab, the simulations were conducted using Autodesk CFD to confirm the principle of this mixer. The results showed that the flow lines in the main chamber were different between expansion and contraction, confirming the asymmetry of the flow, which is the stirring principle of this mixer. However, the flow generated by the continuous repetition of expansion and contraction was complex and difficult to reproduce by simulation. Experiments are necessary to confirm the flow that is actually generated. Therefore, this study was not based on simulation but on experiments.

### 2.3. Experimental Procedure

**Figure 4:**
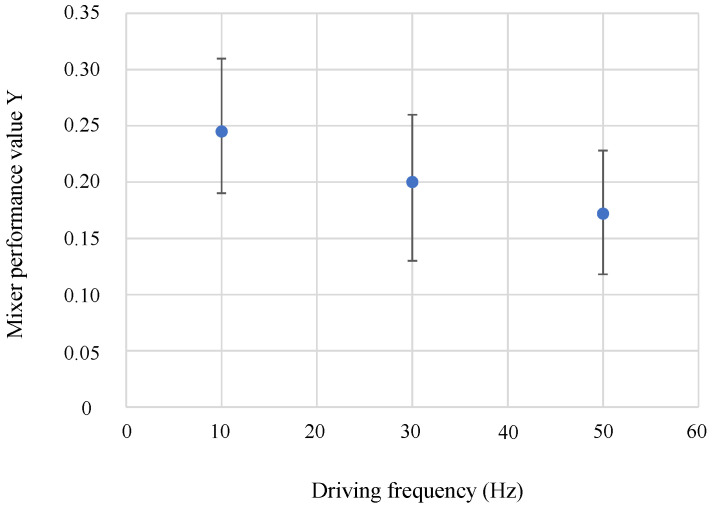
Results of numerical evaluation of mixing performance at the different driving frequencies. The driving pressure is 0.15 MPa, the driving time is 3 s, and the shifting length X of the neck channel is 0 µm.

The experiments were conducted in two steps. First, an experiment was conducted to compare the mixing performance at different neck channel connection positions. In this study, we focused on the amount of deformation of the silicone wall during mixer driving and designed the aspect ratio of the wall to enable mixing in large Sidewall-Driven Micromixers. However, as shown in Figure 1c, the deformation of the silicone wall impedes flow along the wall of the main chamber, resulting in poor mixing performance. Hence, it is considered that the flow impeded by wall deformation can be reduced by shifting the position of the neck channel from the tangential portion of the main chamber, as shown in Figure 3. By conducting an experiment to compare the mixing performance of mixers with various shifting lengths, we determined the appropriate position of the neck channel to maximize the mixing performance. The experiment was conducted at two different driving pressures (0.15 and 0.2 MPa) to determine the change in the appropriate position of the neck channel with respect to different wall deformations. Once, the drive frequency is mentioned. As this device creates a swirling flow by the inequality of push–pull, the mixing performance improves as the driving frequency is increased for the same amplitude. However, as the driving frequency is increased, the amplitude attenuates. Then, a preliminary experiment was conducted to compare the mixing performance at the different driving frequencies. The result is shown in Figure 4. The shifting length X of the neck channel is 0 µm, the driving pressure is 0.15 MPa, and the driving time is 3 s. Although the method for calculating the mixing performance value *Y* is explained in detail in the following paragraph, the smaller the value of *Y*, the better the mixing performance. To improve reproducibility, three mixers of the same dimensions are fabricated, and the value of *Y* is calculated for each. The average values are plotted and the minimum and maximum values are indicated by error bars. The experiment showed that the mixing performance improved up to 50 Hz, which is the limitation of the solenoid valve’s specifications. It was confirmed that the mixing performance improved when the driving frequency was increased, even when the amplitude attenuation was taken into account. Therefore, in this study, the mixer was driven at 50 Hz.

**Figure 5:**
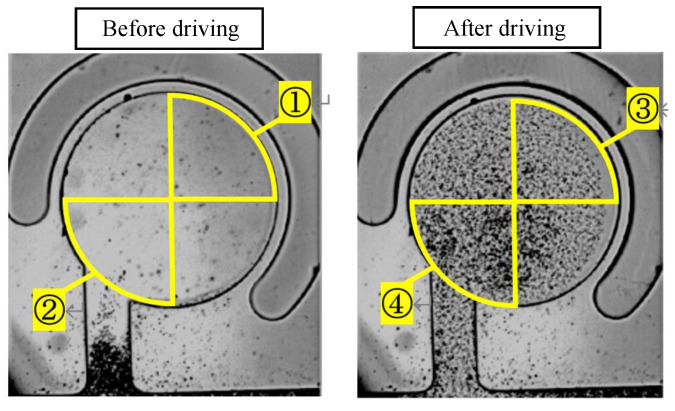
State of a mixer before driving and after driving. Areas 1, 2, 3, and 4 are the segmented regions for luminance evaluation.

The numerical method for evaluating the mixing performance involved comparing the microbead concentrations in the two regions after the mixer was driven. Figure 5 shows the state of the mixer before and after driving.

The average luminances of areas 1, 2, 3, and 4, shown in Figure 4, are determined as *a*, *b*, *c*, and *d*, respectively. The mixing performance value *Y* is defined by Equation (1):

(1) Y=c/a−d/b

The average luminance values of the two areas after mixer driving were divided by the luminance values before mixer driving and normalized. The difference between them was used to evaluate the mixing performance. The smaller the mixing performance value *Y* was, the faster the microbeads reached the wall of the main chamber. Thus, the mixer was evaluated to exhibit excellent mixing performance.

Next, an experiment was conducted to measure the amount of wall deformation during the mixer driving. By comparing the results with the experimental results of mixing performance, we confirmed the relationship between the appropriate connection position of the neck channel and the degree of wall deformation of the mixer. Similar to the experiment involving comparison of the mixing performance, this experiment was conducted at two different driving pressures.

## 3. Result

### Comparison of Mixing Performance

**Figure 6:**
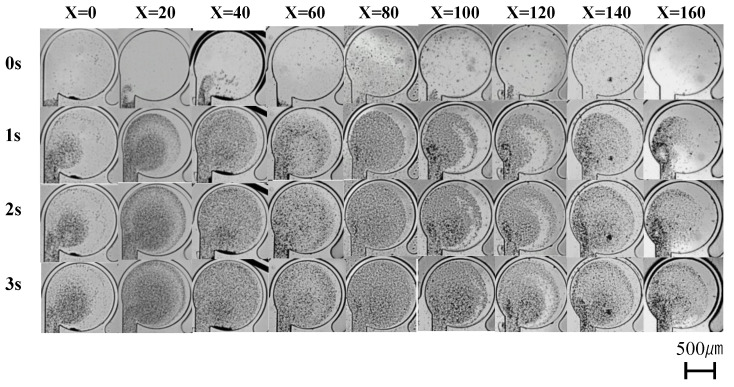
State of mixers during driving. Shifting length is from 0 to 160 µm. Driving pressure is 0.15 MPa, driving frequency is 50 Hz, and driving time is 3 s.

**Figure 7:**
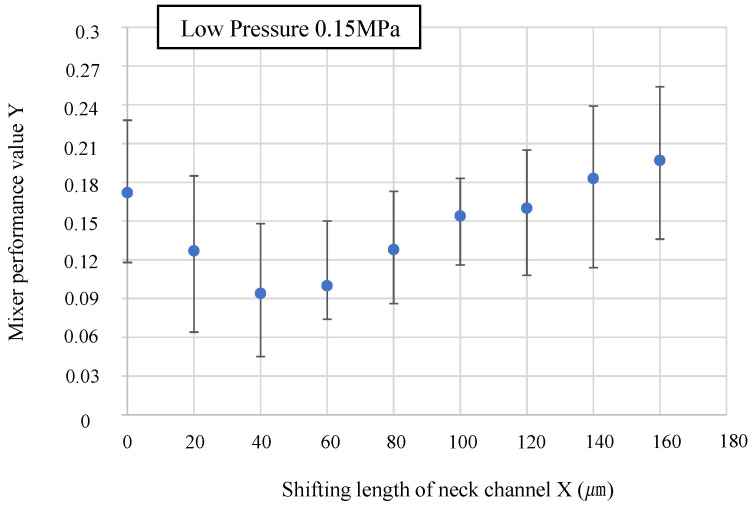
Results of numerical evaluation of mixing performance. At driving pressure of 0.15 MPa, the mixing performance is maximized at shifting length of 40 µm.

Figure 6 shows the condition of the mixer with respect to various neck channel shifting lengths, ranging from 0 to 160 µm, during driving. The driving pressure is 0.15 MPa, the driving frequency is 50 Hz, and the driving time is 3 s. Figure 6 shows that for the mixer without shifting the neck channel (0 µm), the microbeads are concentrated near the neck channel (lower left portion of the main chamber). The flow along the wall is extremely poor, and the microbeads cannot reach the wall. By increasing the shifting length, the flow velocity along the wall increases, the microbeads can reach the wall, and the mixing performance is improved. Shifting the position of the neck channel reduces the impeding flow along the wall owing to wall deformation. However, the mixing performance reaches its maximum at a shifting length of 40 µm, after which it gradually decreases. The state of the mixers with shifting lengths of 120 µm and 140 µm in Figure 6 shows that when the shifting length is increased excessively, a swirling flow is generated on the left side of the neck channel, and the swirling flow on the right side is relatively weakened, which reduces the mixing performance. Figure 7 shows the results of the numerical evaluation of the mixing performance. The method for calculating the mixing performance *Y* is shown in Section 2.3. To improve reproducibility, three to five mixers of the same dimensions are fabricated, and the mixing performance value *Y* is calculated for each. The average values are plotted. Given the variations in the height of SU8-3050 during the production of the mold, mixer channel heights in the range of 290–310 µm are used in the experiments. The error bars indicate the minimum and maximum values of the calculated *Y*.

As shown in Figure 7, the numerical evaluation indicates that the mixing performance reaches its maximum at a shifting length of 40 µm. After this, the mixing performance gradually decreases. Based on these results, at a driving pressure of 0.15 MPa, the appropriate shift length of the neck channel is 40 µm. The same procedure is used to evaluate the effect of increasing the driving pressure of the mixer.

**Figure 8:**
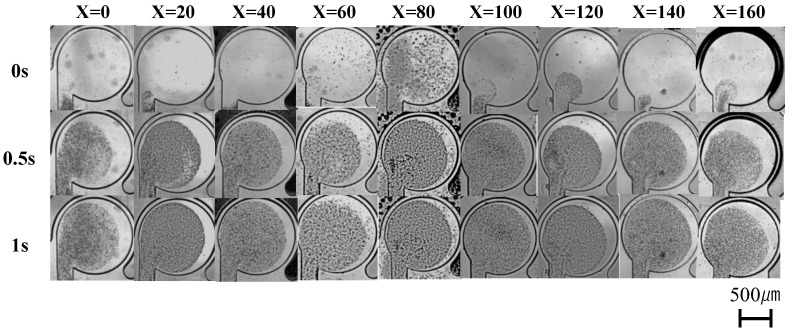
State of mixers during driving. Shifting length is from 0 to 140 µm. Driving pressure is 0.2 MPa, driving frequency is 50 Hz, and driving time is 1 s.

**Figure 9:**
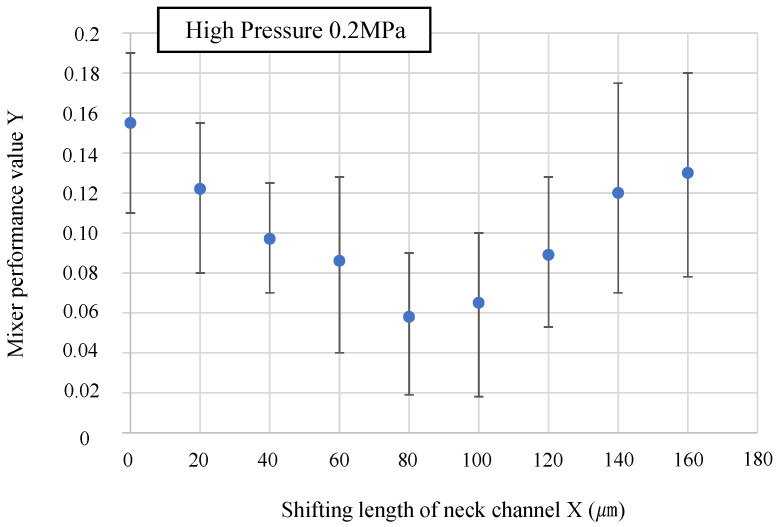
Results of numerical evaluation of mixing performance. At driving pressure of 0.2 MPa, the mixing performance is maximized at shifting length of 80 µm.

Figure 8 shows the state of the mixer during driving when the driving pressure was increased. The driving pressure is 0.2 MPa, the driving frequency is 50 Hz, and the driving time is 1 s. The mixer was identical to that used in the low-pressure experiment (0.15 MPa). Figure 9 shows the results of the numerical evaluation of the mixing performance.

Figure 8 shows that when driven at a high pressure (0.2 MPa), the concentration of microbeads near the neck channel does not occur in any of the mixers, and the flow velocity along the wall is sufficiently fast. However, the mixing performance varied with the shifting length of the neck channel. Figure 9 shows that, when driven at a high pressure (0.2 MPa), the mixing performance reaches its maximum at a shifting length of 80 µm, after which the mixing performance gradually decreases. This result indicates that when the mixer is driven at a higher pressure, the mixing performance can be maximized by increasing the shifting length when compared with that at low pressure. Given that the amount of wall deformation increases when the mixer is driven at high pressure, it is necessary to further increase the shifting length of the neck channel to prevent the flow along the wall from being impeded. To confirm the specific relationship between the neck channel position that maximizes the mixing performance and the amount of wall deformation, an experiment was conducted to measure the amount of wall deformation when the mixer is driven.

## 4. Discussion

**Figure 10:**
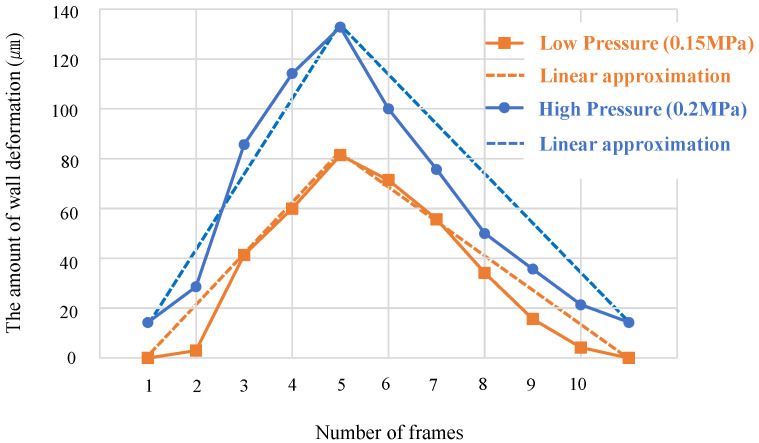
Amount of wall deformation during mixer driving (transition of wall deformation per cycle). The dotted line is a linear approximation for calculating the average wall deformation.

Figure 10 shows the measured wall deformation during mixer driving.

The graph in Figure 10 shows the wall deformation measured by taking 10 frames per cycle of the mixer driven at 50 Hz. Table 1 lists the average wall deformation during mixer driving and appropriate shift length of the neck channel, as previously described. The average wall deformation at low pressure (0.15 MPa) was calculated using a linear approximation connecting a maximum value of 81 µm and minimum value of 0 µm. The average wall deformation at high pressure (0.2 MPa) was calculated using a linear approximation connecting the maximum value of 133 µm and the minimum value of 14 µm.

Table 1 indicates that the average wall deformation during mixer driving and appropriate shift length of the neck channel are almost equal. In a strict sense, these values are not exactly equal because the shifting length of the neck channel, in the experiment that evaluates the mixing performance, is a discrete value with an interval size of 20 µm. However, the discrete value closest to the average wall deformation is the appropriate shift length of the neck channel.

Therefore, the mixer can be optimized as follows. The dimensions of the mixer and height of the silicone wall were determined based on the size of the cells to be inserted. Then, the width of the silicone wall that yielded the largest possible aspect ratio was determined. Next, we determined the pressure required to drive the mixer and conducted a preliminary experiment using a mixer with no neck channel shifting to measure wall deformation. The appropriate position of the neck channel was determined based on the average wall deformation, and a mixer was created. Thereafter, the mixer was optimized by slightly changing the pressure from the initial setting value to determine the pressure at which mixing performance was optimal. There is another optimization method. Owing to the high driving frequency of this mixer, when the driving pressure increased, the wall deformation did not return to zero even when no pressure was applied in the middle of the periodic motion. However, if this is assumed to be zero because it is small when compared to the wall deformation for one cycle, the wall deformation in the static pressure is measured via a simulation that can be easily implemented, and thereby, the shifting length of the neck channel can be determined to be half of the measured maximum wall deformation. This procedure can also be used to optimize the mixer.

## 5. Conclusions

In instances where a large mixer designed for spheroid insertion is driven, the silicone wall’s deformation impedes the flow along the main chamber’s wall, leading to suboptimal mixing performance. This study aimed to tackle this by designing the mixer’s neck channel to connect at a shifted position from the main chamber’s tangential part. This adjustment reduces flow impedance due to wall deformation, enhancing the mixing performance of large mixers. Furthermore, given the close alignment between the average wall deformation when the mixer is driven and optimal shifting length of the neck channel, the mixer can be optimized by establishing the neck channel’s best position based on the average wall deformation derived from preliminary experiments or simulations.

The study underscored that the wall deformation when the mixer is driven profoundly influences the Sidewall-Driven Micromixer’s mixing performance. Although augmenting the wall deformation increased the mixing performance owing to the enhanced volume suctioned into the main chamber, the flow velocity along the wall was decreased due to the deformation itself. Leveraging this understanding, we will optimize the mixing performance by adjusting the driving chamber’s dimensions and form, both crucial determinants of wall deformation. Our evaluations centered solely on the mixer’s mixing performance, using a liquid infused with microbeads. We are considering using PIV (Particle Image Velocimetry) as a new method of evaluating the mixing performance. Compared to the evaluating method using microbead concentration, this method can measure the flow velocity at the point to be checked inside the main chamber, thus visualizing the state of mixing. It is worth noting that we did not execute experiments using actual spheroids or their analogs within the main chamber. In future endeavors, we aim to undertake comprehensive experiments deploying spheroid-sized analogs. For instance, a predefined liquid volume can be introduced from the main channel into the main chamber already containing the spheroid analog, followed by the liquid’s extraction from the main channel. After this, the mixer is activated to agitate a certain volume of liquid and spheroid simulants in the main chamber, and its mixing performance can be assessed. In this case, PIV, described above, can be used to measure the flow velocity around a spheroid to visualize how the drug and spheroids are agitated in the main chamber. If this experiment demonstrates satisfactory mixing efficiency, then it becomes feasible to mix a certain drug quantity with the spheroid without necessitating a fresh drug supply from the main channel. This capability enables drug screenings within the mixer to ascertain the effectiveness of a particular drug volume, rendering the Sidewall-Driven Micromixers more practical. Another major issue in the practical application is how to insert the spheroids into the main chamber. We are considering inserting spheroids into the chamber from the neck by tilting a flow channel and using gravity. Since the spheroid has a large mass, it should be insertable with the use of gravity. If this method cannot be applied, we are considering to use centrifugation as an alternative method. In previous studies, small cells have been successfully inserted into the chamber by spinning the mixer in a centrifugal separator. Therefore, we consider that we can also apply this technique to the spheroids.

## Figures and Tables

**Table 1:** Average wall deformation and shifting length that maximizes mixing performance when the mixer is driven at low pressure (0.15 MPa) or high pressure (0.2 MPa).

	Low Pressure (0.15 MPa)	High Pressure (0.2 MPa)
Average wall deformation (µm)	40.5	73.5
Appropriate shift length (µm)	40	80

## Data Availability

The datasets used and/or analysed during the current study are available from the corresponding author on reasonable request.
